# A Case of Pyemia Treated with Sulphite of Soda

**Published:** 1870-04

**Authors:** O. W. Saddler

**Affiliations:** Lake City


ARTICLE XII.

### A CASE OF PYEMIA TREATED WITH SULPHITE OF SODA.

By O. W. SADDLER, M.D., of Lake City, Feb. 7, 1870.

In October, 1868, I was called to see Mrs. H——, a married lady about 35 years of age, and the mother of seven children, two of which were dead. Her health for several years has been subject to great variations; she has what would be called a scrofulous constitution, several times having had suppuration of the lymphatic glands of the neck, and as a result a great depression of the general system, which by proper tonic and alterative treatment would again recuperate; for two years she had been free from the suppuration of these glands.

With this she had had a profuse uterine leucorrhœa all the time, the discharge being muco-purulent. Before the healing of the glands she became pregnant, when the glands healed more rapidly, the leucorrhœa subsided to a great extent, and she became healthier and more fleshy than for a long time before. After her confinement the leucorrhœa returned very bad, and that, with the lactation, reducing her very low, with very troublesome and painful prolapse and anteversion of the uterus, for which she would do nothing but occasionally inject some astringent into the vagina. After weaning the child she improved a little, but the uterine trouble still remained.

About eighteen hours before my visit, while driving some cows out of the yard, she was taken with a severe chill, retired immediately to the house and tried to get warm, but warmth seemed to do no good; the chill continued about one hour, when she felt as much too warm as too cold before. This reaction remained about as long as the chill had done, when the chills returned with severe vomiting. They immediately sent for their family physician, but he not being at home, I was sent for. On arriving at the house, I found the patient in a cold and exhausted state, partly unconscious, hearing nothing said without it was spoken very loud, and then seeming not to understand—rolling her head slowly and groaning. When her attention was secured, which could be done occasionally, she said her head ached very hard, and she was sore all over—"don't touch me." She was also unable to move her limbs at all. The pulse was very weak and rapid; could not count them accurately; must have been 150 or 160 per minute. Her tongue was dry and a little brown, and some sordes on the teeth. There was also some diarrhœa, of a brown, soft-soapy looking matter, with tenderness over the *centre* of the abdomen and close to the pubis.

I supposed I had a very severe case of typhoid fever, which might produce death almost immediately. I went to work to bring about reaction and stop the vomiting, by putting heat to the body, friction to the extremities, and giving morphia sulph. gr. $\frac{1}{4}$, in peppermint-water, as often as it should be thrown up, or every two hours if not thrown up, followed with quinia sulph. gr. v. and aromatic sulph. acid, m. v., alternately with the morphia to be kept up till reaction was restored, when the same remedies were to be given every four hours alternately.

On visiting her the next morning, I found that reaction was established. It came slow, but no chills were felt after that. She had slept some, had vomited but two or three times, and but one movement of the bowels; the soreness of the muscles still remained, and the same inability to move, and great trouble to swallow—tongue had now become very dry and brown, and a great deal of sordes on the teeth. Continued morphine sufficient to produce rest, and gave sulphite of soda, gr. x., every three hours. Pulse about 100 and stronger.

*Third day.* Found sordes thicker and covering her lips inside and out, also the roof of her mouth, and inside of her cheeks, unable to taste the difference between bitter and sweet, the only sensation being a smarting produced by any and all liquids alike, except acids, which were worse. Consciousness wholly returned, except at night, when she was somewhat delirious, yet unable to follow a line of conversation. Her diet consisted of beef and mutton teas, and wine. No vomiting; but one operation of the bowels; pulse about the same as yesterday. Continued the sulphite the same as before, and morphia at night.

*Fourth day.* Sometime during the evening or night, a large abscess broke, and evacuated itself through the vagina. This morning she is more quiet; the soreness is all gone from the flesh, and she can swallow better and move a little. The scabs are thicker, darker, and dryer than yesterday. Put glycerine on the outside, and gargled the mouth with a weak solution of carbolic acid. Continued other treatment as before.

*Fifth day.* Rested very well last night; feels better this morning, and says her head feels natural again. This morning she is poking the scabs out of her mouth with her tongue, and has cleaned her teeth, looking in a small glass to guide her work by; very weak, and can pick them and her lips but a little while at a time. Continued sulphite, and whatever she chooses for nourishment, as she now has a little appetite.

*Sixth day.* Mouth and lips all cleaned off; eyes look bright; appetite still better. Gave seidlitz powder as laxative; continued sulphite.

*Tenth day.* Found her sitting up and dressed. The cause of all this trouble, I consider to be the abscess in the pelvis, and the highly poisoned state of the blood, from the absorption of pus from the same, the exact location of which I was unable to determine, as she would not permit an examination to be made. Its obscurity arose from there being no febrile action connected with its formation, and no uncommon local pain, not enough at least to make them call my attention to it.

Now, I do not claim that the sulphite saved the woman's life, for I think that had not the abscess broke, the absorption of pus would have continued to be more rapid than the sulphite could have neutralized, but the blood being so thoroughly saturated with it, when exit was given to the supply of pus the recovery was very rapid.

